# Pediatric Rapunzel Syndrome Presenting With Jejunojejunal Intussusception Managed Surgically: A Rare Case Report

**DOI:** 10.1002/ccr3.71634

**Published:** 2025-12-10

**Authors:** Muhammad Irfan, Hafiz Abdul Mughees, Muhammad Talha Safdar, Muhammad Hassaan Javaid, Rehman Asif, Abubakr Mahmoud, Muhammad Bilal Masood, Tashfeen Farooq, Fatima Nazir

**Affiliations:** ^1^ Department of Surgery Shaheed Mohatarma Benazir Bhutto Medical College Lyari Karachi Pakistan; ^2^ Department of Surgery Foundation University Medical College Islamabad Pakistan; ^3^ Shifa College of Medicine Islamabad Pakistan; ^4^ Shaheed Mohatarma Benazir Bhutto Medical College Karachi Pakistan; ^5^ Faculty of Medicine University of Khartoum Khartoum Sudan; ^6^ Department of Internal Medicine Wah Medical College Wah Pakistan; ^7^ Department of Surgery Advance International Hospital Islamabad Pakistan; ^8^ Department of Surgery Rawalpindi Medical University Rawalpindi Pakistan

**Keywords:** jejunojejunal intussusception, pediatric abdominal mass, pediatric surgery, Rapunzel syndrome, trichobezoar, trichotillomania‐associated complications

## Abstract

Bezoars are masses of indigestible material within the gastrointestinal tract. Trichobezoars, composed of hair, are rare and typically occur in young females with psychiatric conditions. Rapunzel syndrome is a severe variant where the trichobezoar extends into the small intestine. We report a case of a 7‐year‐old girl presenting with acute abdominal pain, reduced appetite, and a palpable epigastric mass. She had a history of trichophagia and weight loss. Investigations revealed microcytic hypochromic anemia and a large intragastric mass extending into the duodenum, with associated jejunojejunal intussusception on CT scan. She underwent laparotomy with gastrotomy, and a 15 × 7 cm trichobezoar was removed. Intraoperatively, the intussusception had spontaneously reduced, and the small bowel was viable with no resection required. Postoperative recovery was complicated by wound infection. Psychiatric assessment confirmed trichotillomania and trichophagia. This case emphasizes the importance of early recognition of Rapunzel syndrome in children with behavioral symptoms and abdominal complaints. Surgical removal remains the definitive treatment, and psychiatric follow‐up is crucial to prevent morbidity and recurrence. Prompt diagnosis, timely surgical management, and structured psychiatric follow‐up are essential to prevent recurrence and morbidity.

## Key Clinical Message

Rapunzel syndrome is a rare pediatric disorder and a rare cause of jejunojejunal intussusception in young children. Early detection through history and imaging enables timely surgical intervention preventing complications. Psychiatric assessment and long‐term follow‐up are crucial to avert recurrence, in pediatric patients with Trichotillomania and trichophagia.

## 1 Introduction

A Bezoar is an indigestible material that collects in the gastrointestinal (GI) tract, forming a hard mass and is caused by both food as well as non‐food material. Based on the content, there are four primary types of Bezoars [1]. Phytobezoars are a conglomerate of fibers of vegetable and fruit; the most common type of Bezoar [2]. Trichobezoars are composed of hair and are usually black regardless of the patient's hair color [3]. Pharmacobezoars are composed of medicines, and Lactobezoars are composed of undigested milk and mucus, and typically occur in infants [4]. In addition, some don't fall under these four categories and are called other types of bezoars, which are composed of undigested chewing gum, plastic, paper, etc. [5]. The incidence of bezoars varies with the geography and culture because dietary habits differ across regions [6]. Bezoars are asymptomatic or usually show no specific symptoms until they enlarge enough to cause serious complications such as intussusception, perforation, appendicitis, and peritonitis etc. [7].

Rapunzel Syndrome is a rare form of trichobezoar. This condition arises as the gastric bezoars grow bigger, extending into the small intestine and forming long, tail‐like hair projections [8]. It can manifest at any age with marked predominance in females, especially those with psychotic disorders and developmental delay [9]. Trichobezoar is found to have a strong association with trichotillomania, characterized by compulsive hair‐pulling behaviors, and trichophagia, characterized by ingestion of hair [10].

## 2 Case Presentation

A 7 year old female child, described as socially active, presented to the outpatient department with complaints of acute‐onset, severe generalized abdominal pain, first noted 2 days prior to evaluation. The pain was stabbing in nature, rated 10 out of 10 in intensity, and occurred in intermittent episodes, each lasting approximately 1 h. The discomfort was consistently exacerbated by oral intake, particularly solids, and was partially relieved by analgesics. The pain did not radiate. Additional symptoms included a reduced appetite over the preceding 48 h, during which she limited her intake to fluids, avoiding solid food. The clinical history further revealed chronic trichophagia and unintentional weight loss, though the exact duration was not clearly established. There was no significant past medical or surgical history.

On physical examination, the patient was alert and well‐oriented to person, place, and time. Vital signs were within normal limits. There were no observable signs of pallor, icterus, peripheral edema, digital clubbing, or lymphadenopathy. Abdominal assessment revealed a non‐distended abdomen with a firm and tender mass palpable in the epigastric region. Bowel sounds were audible and normal in character. The rest of the examination findings were unremarkable. There were no signs of peritonitis.

### 2.1 Investigations

Initial laboratory evaluation revealed microcytic hypochromic anemia, with a hemoglobin level of 10.2 g/dL and a mean corpuscular volume (MCV) of 64.2 fL, consistent with iron deficiency anemia. The total leukocyte count was elevated at 11.60 × 10^3^/μL, and the platelet count was 502,000/μL. Serological testing for hepatitis B surface antigen (HBsAg) and hepatitis C virus (HCV) was non‐reactive. Blood cultures showed no growth.

**FIGURE 1:**
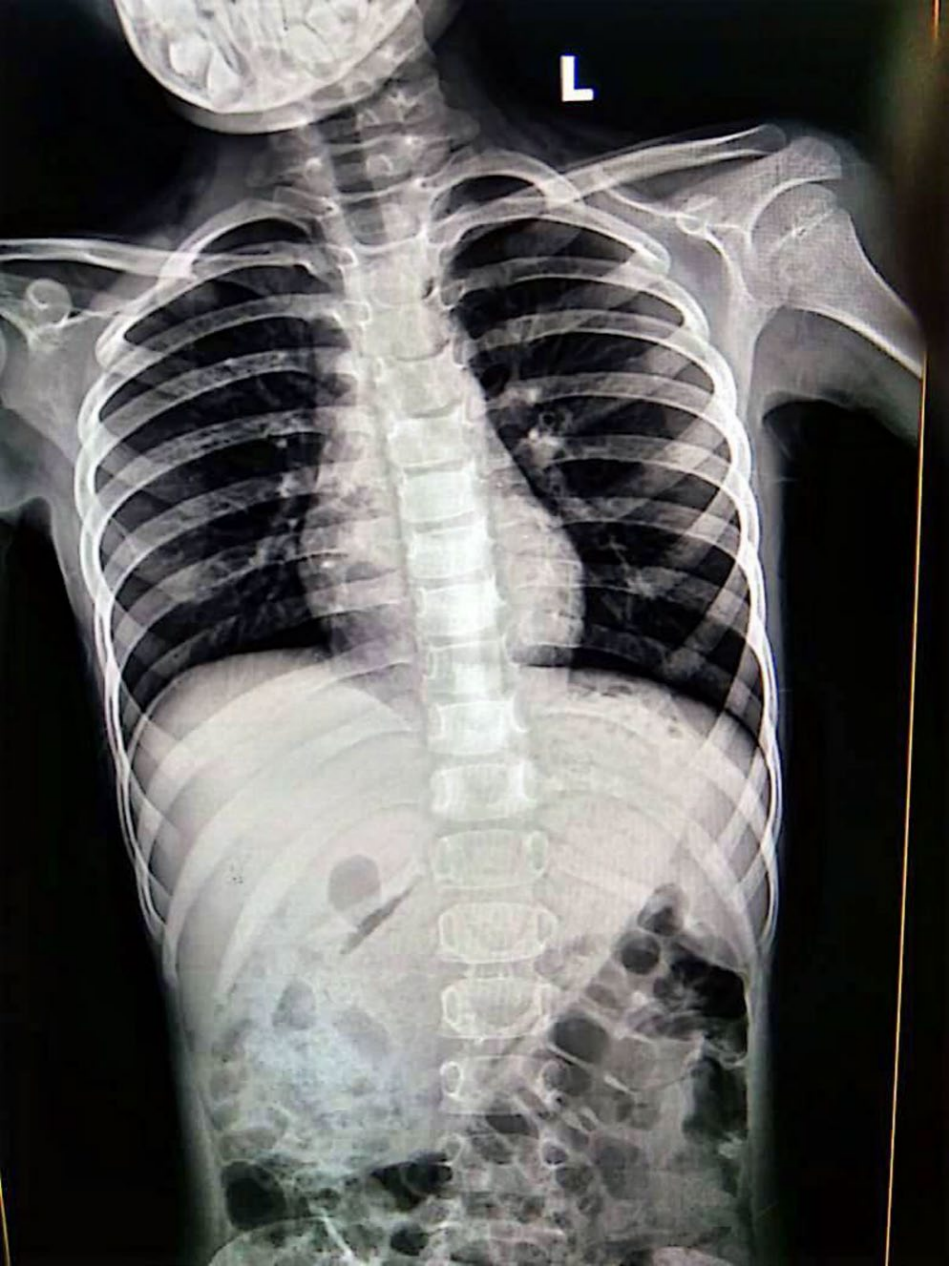
Anterior aspect X‐ray chest and abdomen showing a radio opaque object within stomach and small bowel obstruction.

**FIGURE 2:**
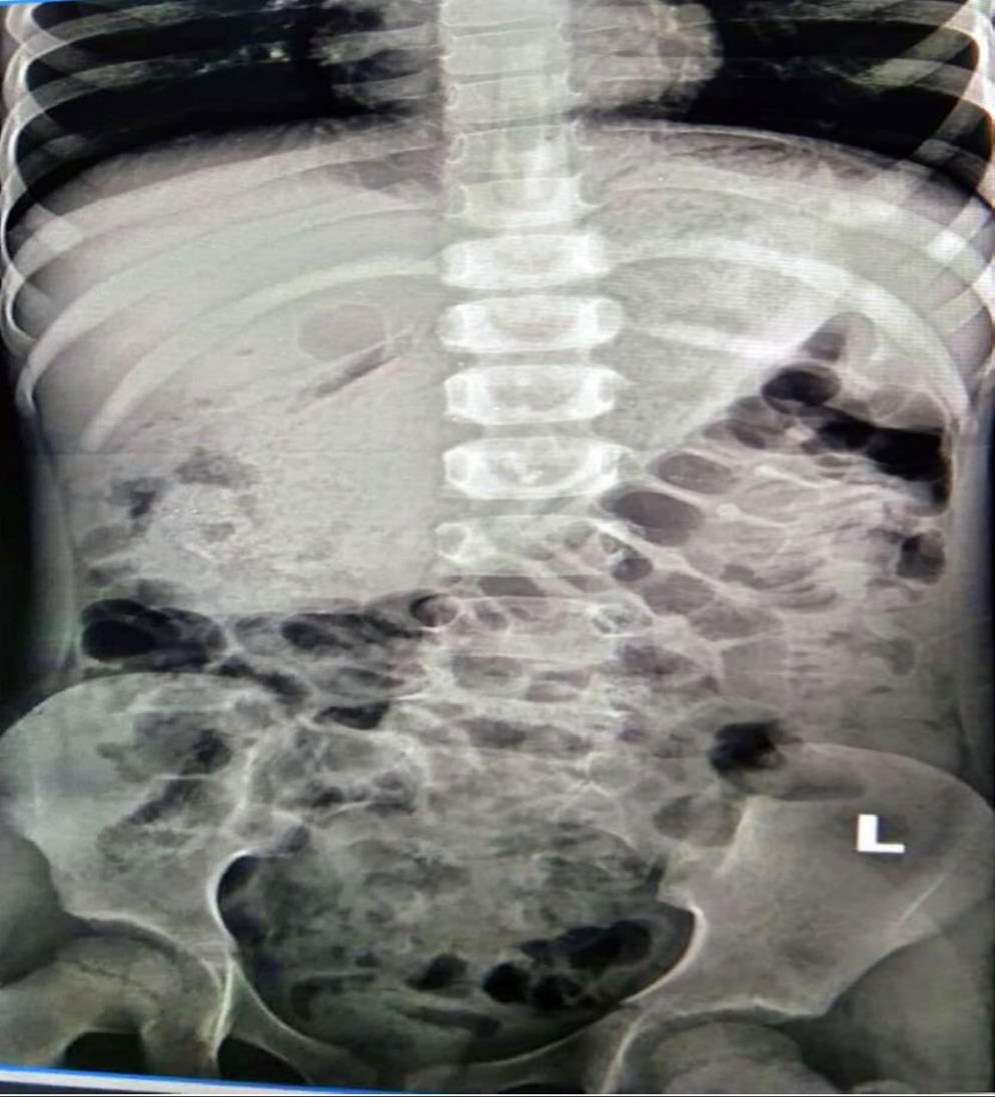
Posterior aspect of X‐ray chest and abdomen showing similar findings.

Abdominal X‐ray radiography shown in Figures 1 and 2 demonstrated a radio‐opaque shadow within the stomach, displacing adjacent bowel loops inferiorly, suggestive of a large intragastric mass. Ultrasonography of the abdomen showed mild hepatomegaly with homogeneous echotexture and no evidence of focal lesions or abscess formation. Additionally, a few enlarged mesenteric lymph nodes were noted, likely representing reactive inflammatory changes.

**FIGURE 3:**
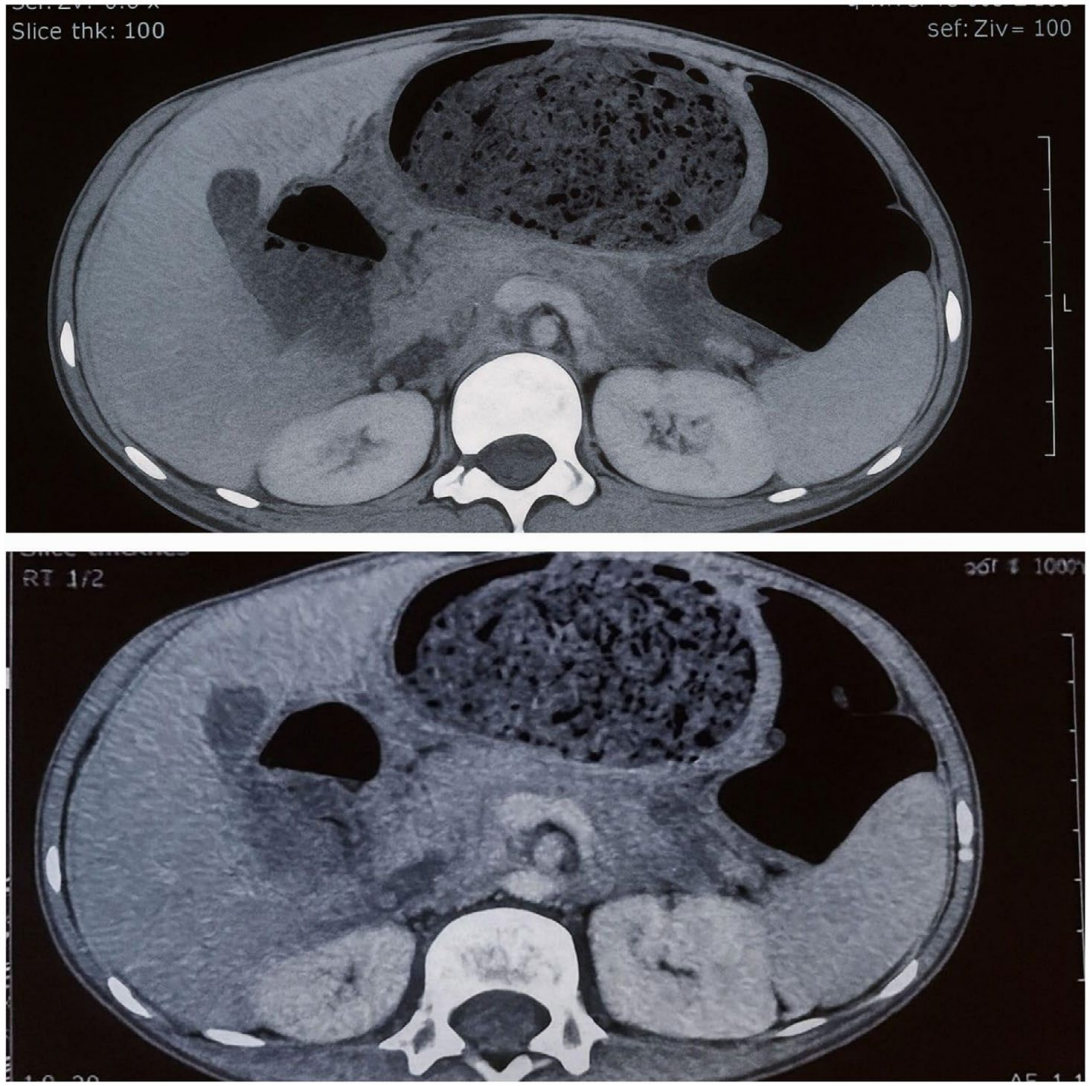
Contrast‐enhanced computed tomography (CT) of the abdomen showing large gastric tracheibezoar as well as jejunojejunal intussusception with mesenteric lymphadenopathy.

Further evaluation with contrast‐enhanced computed tomography (CT) of the abdomen, shown in Figure 3, confirmed the presence of a large gastric bezoar consistent with Rapunzel syndrome. The CT also revealed jejunojejunal intussusception with mesenteric lymphadenopathy serving as the probable lead point. There was no evidence of intestinal obstruction or pneumoperitoneum. Based on the imaging and clinical findings, surgical referral was advised for definitive management.

### 2.2 Treatment

**FIGURE 4:**
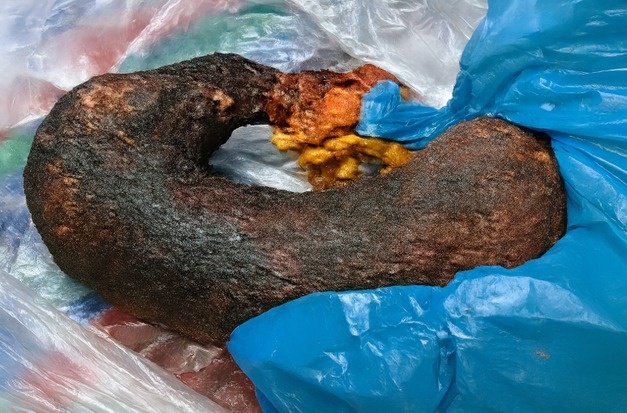
Trichobezoar extracted as a single piece measuring 15 × 7 cm which had filled the stomach and extended into the abdomen.

Based on imaging, a decision was made to perform laparotomy and gastrotomy. Laparotomy was preferred over laparoscopy due to the size and extent of the bezoar. A 5 cm gastrotomy incision was made on the anterior wall of the stomach, through which the trichobezoar was successfully extracted as a single mass. The trichobezoar shown in Figure 4 measured 15 × 7 cm and had filled the stomach, extending into the duodenum. During surgery it was noted that the jejunojejunal intussusception, which was revealed on the CT scan had spontaneously reduced. The entire small bowel was carefully inspected from the duodenojejunal flexure to the ileocecal junction. There was normal color and vascularity. No ischemia, perforation or bowel resection was identified. Due to which bowel resection was not required. The surgery was successful with no intraoperative complications.

### 2.3 Follow‐Up and Postoperative Management

Psychiatric evaluation diagnosed her with trichotillomania, trichophagia, and anxiety. She was referred for ongoing psychiatric follow‐up and support to prevent the recurrence of the condition.

**FIGURE 5:**
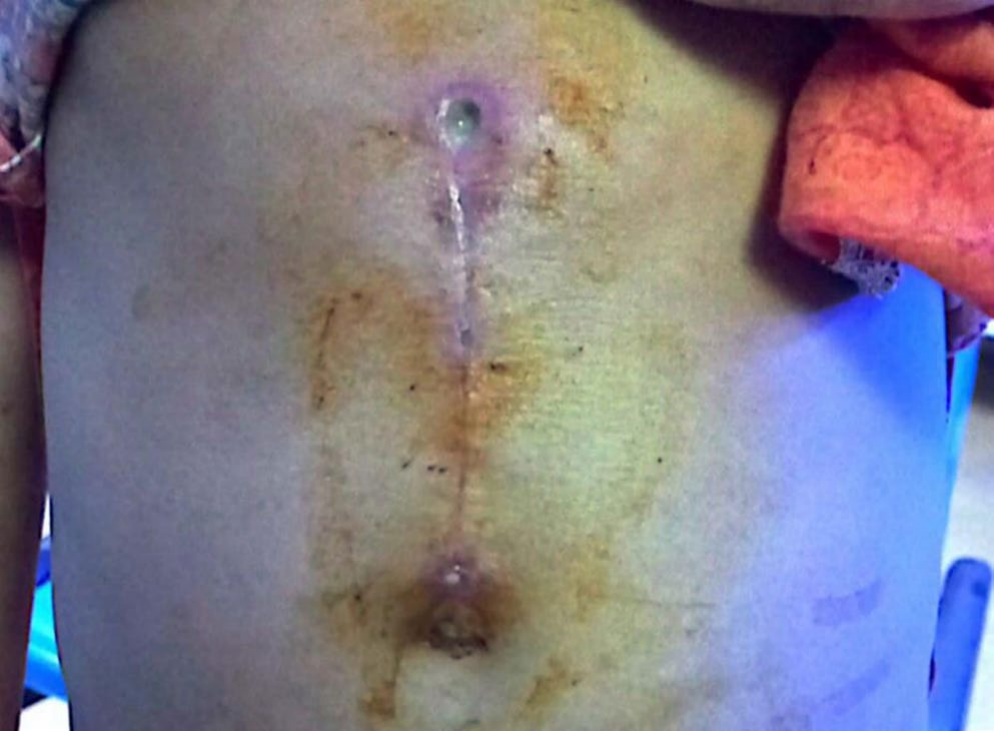
Postoperative infected scar due to infection at surgical site.

She developed an infected wound with purulent discharge at the surgical site shown in Figure 5, requiring additional wound care and management. The patient was kept on an injection of aminoglycosides 115 mg twice daily for 7 days along with daily dressing. She was discharged with a multivitamin syrup for 1 month to boost her nutritional support.

## 3 Discussion

Bezoars are formed by the accumulation of indigestible materials and chemicals within the gastrointestinal tract, leading to the development of a compact mass or concretion [11]. Rapunzel syndrome is a rare variant that arises when a trichobezoar extends beyond the stomach into the small intestine due to the habitual ingestion of hair [12]. Common clinical manifestations include nausea, vomiting, abdominal pain, anorexia, constipation, diarrhea, weight loss, and features of malnutrition [13]. The specific symptoms of Rapunzel syndrome can vary based on the size and location of the trichobezoar within the gastrointestinal tract [14]. In cases such as ours, the elongated tail of the trichobezoar projecting into the small intestine may act as a pathological lead point for intussusception [15]. However, in this case, the jejunojejunal intussusception identified on CT imaging was found to have spontaneously reduced during surgery. The bowel was viable on inspection requiring no resection. This aligns with a few reports in the literature describing transient intussusception due to traction from the bezoar tail.

A comprehensive review of the literature identified 108 documented cases of trichobezoars, the majority of which occurred in female patients, consistent with our case [13]. Trichobezoars are predominantly observed in individuals between 10 and 20 years of age. However, the distinct feature of this case lies in the presentation in a 7‐year‐old girl [16]. Rapunzel syndrome is frequently associated with underlying behavioral or psychiatric disorders. In this case, the patient had a chronic history of trichophagia that led to the formation of a large gastric trichobezoar. However, unlike the majority of reported cases, no formal psychiatric diagnosis had been established, and no prior psychiatric intervention was undertaken, making this presentation notably distinct [17]. Trichobezoars can vary significantly in size, ranging from small masses measuring approximately 2 × 2 cm to large ones reaching up to 30 × 15 × 10 cm. In the current case, the trichobezoar measured 15 × 7 cm, classifying it as a large gastric trichobezoar [18].

As the condition advances, initially silent symptoms often evolve into vague, nonspecific complaints, making early diagnosis difficult. Early recognition is particularly important, as obstructive bezoars may lead to serious complications, including gastrointestinal ulceration, bleeding, perforation, and pressure‐induced necrosis [19]. In this reported case, early diagnosis was achieved without the use of endoscopy, commonly considered the gold standard for identifying trichobezoars. Initial evaluation with plain abdominal radiography and ultrasonography suggested an abnormal intra‐abdominal mass, and CT confirmed the presence of a trichobezoar along with associated jejunojejunal intussusception. This emphasizes the diagnostic value of imaging modalities, particularly CT, in cases where endoscopy cannot be awaited, allowing for prompt diagnosis and timely surgical intervention [20].

This emphasizes that cross‐sectional imaging not only aids in identifying bezoars but can also detect secondary complications such as intussusception or obstruction, guiding appropriate surgical planning and timely intervention. Surgical intervention was prompt and effective. The patient underwent exploratory laparotomy followed by anterior wall gastrotomy for complete removal of the trichobezoar. While laparoscopic techniques are increasingly being employed for bezoar extraction, open surgery remains the standard approach in cases involving large or complex bezoars, as it allows for better visualization, safer removal, and assessment of any associated complications such as bowel involvement or intussusception [21].

## 4 Conclusion

Rapunzel syndrome is a rare pathological condition that can cause serious health risks often related to trichotillomania and trichophagia.

Physicians must understand the risk factors that contribute to the progression of trichobezoar. The final diagnosis can be reached with the aid of proper history, examination, and early investigation. The gold standard intervention for this condition is laparotomy. In order to prevent recurrence, individuals with trichobezoar should be kept under surveillance by psychiatrists as part of their treatment plan.

## Author Contributions

**Muhammad Irfan:** conceptualization, project administration. **Hafiz Abdul Mughees:** conceptualization, formal analysis, methodology, visualization, writing – review and editing. **Muhammad Talha Safdar:** conceptualization, methodology. **Muhammad Hassaan Javaid:** conceptualization, formal analysis, methodology. **Rehman Asif:** conceptualization, methodology. **Abubakr Mahmoud:** conceptualization, methodology. **Muhammad Bilal Masood:** conceptualization, methodology. **Tashfeen Farooq:** conceptualization, methodology. **Fatima Nazir:** conceptualization, methodology.

## Funding

The authors have nothing to report.

## Ethics Statement

This case report was conducted in accordance with the Declaration of Helsinki. Ethical approval was obtained from the Institution.

## Consent

Written informed consent was obtained from the patient's guardian for publication.

## Conflicts of Interest

The authors declare no conflicts of interest.

## Data Availability

Data sharing is not applicable to this article as it is a case report and no datasets were generated or analyzed during the current study.
